# New insights into the QuikChange^TM^ process guide the use of Phusion DNA polymerase for site-directed mutagenesis

**DOI:** 10.1093/nar/gku1189

**Published:** 2014-11-15

**Authors:** Yongzhen Xia, Wenqiao Chu, Qingsheng Qi, Luying Xun

**Affiliations:** 1 State Key Laboratory of Microbial Technology, Shandong University, Jinan 250100, P. R. China; 2 School of Molecular Biosciences, Washington State University, Pullman, WA 99164-7520, USA

## Abstract

The QuikChange^TM^ site-directed mutagenesis method is popular but imperfect. An improvement by using partially overlapping primers has been reported several times; however, it is incompatible with the proposed mechanism. The QuikChange^TM^ method using complementary primers is proposed to linearly amplify a target plasmid with the products annealing to produce double-stranded DNA molecules with 5′-overhangs. The overhang annealing is supposed to form circular plasmids with staggered breaks, which can be repaired in *Escherichia coli* after transformation. Here, we demonstrated that the PCR enzyme fills the 5′-overhangs in the early cycles, and the product is then used as the template for exponential amplification. The linear DNA molecules with homologous ends are joined to generate the plasmid with the desired mutations through homologous recombination in *E. coli*. The correct understanding is important to method improvements, guiding us to use partially overlapping primers and Phusion DNA polymerase for site-directed mutagenesis. Phusion did not amplify a plasmid with complementary primers but used partially overlapping primers to amplify the plasmid, producing linear DNA molecules with homologous ends for site-directed mutagenesis.

## INTRODUCTION

Polymerase chain reaction (PCR)-based site-directed mutagenesis is an essential technique in molecular, biochemical and genetic studies. Several methods have been developed, including overlap extension PCR, megaprimer PCR, QuikChange^TM^ site-directed mutagenesis (1–5), among which the QuikChange^TM^ method (Agilent Technologies, La Jolla, CA, USA) is widely used (6). According to its specification, the QuikChange^TM^ method uses PfuTurbo DNA polymerase to amplify a circular plasmid with a pair of complementary primers, which are completely overlapping to each other (Supplementary Figure S1). The PCR progresses via linear amplification for the production of single-stranded DNA molecules that anneal to generate the circular plasmid with staggered single-stranded DNA breaks (the QuikChange^TM^ manual; Agilent Technologies). The proposed process is similar to a process done in two separate PCR reactions before mixing the products to generate the circle DNA with staggered single-stranded DNA breaks (7). After DpnI digestion of the methylated plasmid template and transformation into *Escherichia coli*, the host cells repair the breaks to yield the plasmid with the desired mutation.

The QuikChange^TM^ manual recommends continuing with transformation even when the PCR product is not detectable via gel electrophoresis. In our experience, the mutation is frequently unsuccessful when the PCR product is undetectable. When this happens, it is hard to troubleshoot. Several labs have also noticed the deficiency and have reported improved success by using partially overlapping primers (4,6,8–10). However, the use of partially overlapping primers should generate linear DNA molecules with short homologous ends, which requires homologous recombination to generate the plasmid with the desired mutations after transformation into *E. coli*. The mechanism is incompatible with the proposed reaction mechanism for the QuikChange^TM^ method (9,10). Here we provide experimental evidence that the QuikChange^TM^ PCR with a pair of complementary primers also produces double-stranded DNA with short homologous ends. After transformation, the PCR products are correctly recombined through an uncharacterized RecA-independent recombination system in *E. coli* cells. Thus, the PCR products are linear double-stranded DNA with homologous ends, whether the reaction is done with completely overlapping primers or partially overlapping primers. The new understanding clarifies the mechanism of a widely used method and explains why using partially overlapping primers are advantageous over complementary primers, which are prone to form primer-dimers. According to the new understanding, we developed a new protocol with Phusion DNA polymerase and partially overlapping primers to generate the PCR products for efficient site-directed mutagenesis.

## MATERIALS AND METHODS

### *E. coli* strains and plasmids

The *E. coli* strains and plasmids are listed in Supplementary Table S1, and primers are given in Supplementary Table S2. *E. coli* XL1-Blue MRF’ was cultured in Lysogeny broth (LB) medium with appropriate antibiotics when needed. The competent cells were prepared in LB broth containing 10% polyethylene glycol 8000 (Sigma), 5% dimethyl sulfoxide and 20 mM MgSO_4_, according to a reported method (11). The competent cell density was adjusted to about 10^9^ per ml. Normally, 5 μl of the non-purified PCR product, or as specified in the text, was used to transform 25 μl of the competent cells, and 250 μl of LB medium was added for recovery at 37°C for 1 h. Then, the sample was spread on LB agar plates with proper antibiotics and reagents. After overnight culture at 37°C, the colonies were counted. Kanamycin (Kan) and ampicillin (Amp) were used at 25 and 100 μg ml^−1^, respectively.

Electroporation, instead of transformation, was used for *E. coli* strains GB05 and GB05dir cells that required induction (12). They were cultured in 50 ml of LB medium containing 0.8% arabinose until their OD_600_ reached 0.5. Then, the cells were collected, and electroporation competent cells were prepared with a density about 10^9^ ml^−1^ (12).

### QuikChange^TM^ site-directed mutagenesis

The QuikChange^TM^ PCR reactions in 20 μl were performed according to the suppliers’ instructions (Agilent). The PCR reactions were also done with commercially available PfuTurbo (Agilent), PrimeStar (Takara), TransTaq (Transgene), Phusion (Fisher-Thermo Scientific), Pyrobest DNA polymerase (Takara), KOD FX DNA polymerase (Toyobo Life Science) and Q5 DNA polymerase (New England Lab) in 20 μl and optimized according to the suppliers’ instructions (Supplementary Figure S2A). The PCR products were treated with DpnI before transforming into the *E. coli* strain XL-1 Blue MRF’ competent cells. The transformed cells were spread onto LB agar plates containing Amp, IPTG and X-Gal. The *E. coli* cells with pBluescript SK- produced blue colonies on the plates (13). The colonies were counted and analyzed. The results were averages of three experiments with standard deviations. PrimeStar DNA polymerase could be used to replace PfuTurbo DNA polymerase for the QuikChange^TM^ site-directed mutagenesis and was used for most of the reactions due to its price advantage.

### Plasmid construction

The pBluescript SK- LacZα region, instead of the MCS region, was selected for mutation. Site-directed mutagenesis was used to convert pBluescript SK- to pBS-TAA (Supplementary Figure S3), which had the stop codon TAA inserted between the codons of the 18th and 19th amino acid residues of the LacZα peptide. The pBS-Kan plasmid contained a Kan-resistant gene inserted between codons for the 18th and 19th amino acid residues (Supplementary Figure S3). The primers used to amplify the Kan-resistant gene from pBBR1MCS-2 (14) and to generate linear pBluescript SK- are listed in Supplementary Table S2. The Kan-resistant gene and linear pBluescript SK- DNA were assembled into pBS-Kan by using the Gibson method (15). The *E. coli* cells with pBS-TAA or pBS-Kan formed white colonies on the agar plates containing IPTG and X-Gal.

### Site-directed mutagenesis using a single-primer PCR method

A single-primer PCR site-directed mutagenesis strategy was also performed. Two PCR reactions were carried out in parallel, and each contained one of the primer pair. PCR conditions were the same as described above for 16 cycles. After DpnI digestion, the two PCR products were combined, mixed and divided equally into two tubes. Then one tube was kept on ice as the control, and the other was heated to 98°C and then slowly cooled to 37°C through a temperature gradient (98°C, 5 min; 90°C, 1 min; 80°C, 1 min; 70°C, 0.5 min; 60°C, 0.5 min; 50°C, 0.5 min; 40°C, 0.5 min; 37°C, holding) to promote annealing. Then 5 μl of the product from each preparation was transformed into the *E. coli* competent cells. The PCR products were also checked with agarose gel electrophoresis.

### Mutate pBS-TAA plasmid and pBS-Kan back to pBluescript SK-

The same pair of primers mutF2 and mutR2 was used to mutate pBS-TAA and pBS-Kan back to pBluescript SK- (Supplementary Figure S3). Reactions were routinely done in a 20-μl volume, and 5 μl of the PCR product was used for transformation. For quantification, the reaction volume was increased to 200 μl. After DpnI digestion, half of the PCR product was separated on agarose gel, and the linear DNA was cut and purified with the E.Z.N.A.™ Gel Extraction Kit (Omega Bio-Tek, USA). The other half of the PCR product was directly purified by using the E.Z.N.A.™ Cycle-Pure Kit (Omega Bio-Tek, USA) to obtain all DNA. The purified DNA products were quantified with a Nanodrop 1000 Spectrophotometer (Thermo Scientific), and 60 ng of DNA was transformed into *E. coli*. The blue/white screening method was used to detect the regeneration of pBluescript SK- (13). The results were averages of three experiments with standard deviations.

### The end filling of the QuikChange^TM^ PCR product

The QuikChange^TM^ PCR with pBS-TAA as the template was done. After DpnI treatment, the product was heated at 80°C for 1 min to linearize any plasmid DNA joint by the primer overhangs, which had a calculated Tm of 72.4°C by using OligoAnalyzer 3.1, a nearest-neighbor thermodynamic method, on IDT website (http://www.idtdna.com/pages/scitools/SciTools) (16). Then the temperature was lowered to 72°C and incubated for 30 s to fill in the end gaps by the PCR enzyme in the PCR reaction. Five microliters of the sample or control without the end-filling step was transformed into *E. coli* to regenerate pBluescript SK-. The results were averages of three experiments with standard deviations.

### Identification of the 5′ ends of the QuikChange^TM^ PCR product

QuikChange^TM^ PCR was done with 5′-phosphorylated primers mutF3 and mutR3. The PCR product was purified with the E.Z.N.A.™ Cycler-Pure kit (Omega Bio-Tek, USA). Then the purified PCR product was ligated by T4 DNA ligase. The ligation product was digested by DpnI to remove the template and purified. The digestion product was diluted for 1000-folds, and the ligation region was amplified by PCR. The PCR product was sequenced with the M13R primer.

### Testing 5′-overhang filling with qPCR melting curve analysis

Four oligonucleotides O1, O2, O3 and mutR2 were used (Supplementary Table S2). O1, O2 and mutR2 at 0.4 μM each were used in a typical PrimeStar PCR reaction as recommended by the supplier (Takara). The reaction went through a single cycle of 98°C for 2.5 min, 61°C for 30 s and 72°C for 3 min. The reaction was stopped by adding ethylenediaminetetraacetic acid (EDTA) to 5 mM (17). After the reaction, the samples were stored on ice. The control contained EDTA before the PCR cycle. Then, 2.5 μl of 20×SYBR Green I solution (Gene-bio, Beijing) was added to 50 μl of sample before the melting curve analysis on a qPCR (Lightcycler^®^ 480 System, Roche). A similar set of experiments was done with the formation of the O1:O2:mutR2 complex (0.4 μM) by heating and cooling in the PCR reaction mixture without the enzyme through a temperature profile of 98°C for 30 s, 88°C for 30 s, 78°C 30 s, 68°C 1 min, 58°C 1 min, 48°C 1 min, 38°C 1 min, 25°C 1 min, and on ice for storage. PrimeStar was a hot-start enzyme and was activated at 98°C for 2.5 min. The activated enzyme was added to the samples, and the reactions were incubated at either 61 or 72°C. EDTA was added to stop the reaction. After SYBR Green I was added, the samples were analyzed.

## RESULTS

### Test high-fidelity DNA polymerases for QuikChange^TM^ mutagenesis

**Figure 1.**
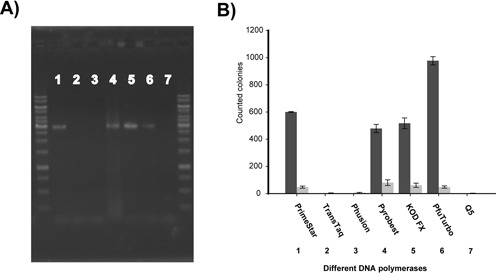
The QuikChange^TM^ method PCR with different high fidelity DNA polymerases. (**A**) Gel electrophoresis of the PCR products produced by different DNA polymerases; 3 μl of each product was analyzed. Lane 1, PCR product of PrimeStar; Lane 2, PCR product of TransTaq; Lane 3, PCR product of Phusion; Lane 4, PCR product of Pyrobest; Lane 5, PCR product of KOD FX polymerase; Lane 6, PCR product of PfuTurbo; Lane 7, PCR product of Q5 DNA polymerase. (**B**) Colonies were formed after transformation of the PCR products. Five microliters of the QuikChange^TM^ PCR product was transformed into *E. coli* XL-1 Blue MRF’ cells; colonies were counted. Dark gray column, blue colonies; Light gray column, white colonies. The data are averages of three experiments with standard deviations (error bar). The lane numbers in (**A**) correspond to the column numbers in (**B**).

Seven high-fidelity DNA polymerases were tested to mutate pBS-TAA back to pBluescript SK-, according to the QuikChange^TM^ protocol. PfuTurbo, PrimeStar, KOD FX and Pyrobest were successful for the mutagenesis, but TransTaq, Phusion and Q5 did not (Figure 1B). When 3 μl of the PCR products was analyzed by using gel electrophoresis, visible bands at 3 kb were detected from the PCR reactions with PfuTurbo, PrimeStar, KOD FX and Pyrobest, but not others (Figure 1A). The clear visible bands suggest exponential instead of linear amplification of the template plasmid pBS-TAA. To confirm the amplification was not linear, the QuikChange^TM^ PCR was separated into two PCR reactions with either the forward primer or reverse primer for the same number of cycles. Then the reaction products were combined and annealed to form double-stranded DNA. However, DNA bands were not detected after gel electrophoresis (Supplementary Figure S4). After transformation, only a few colonies (<5) grew on plates and all of them were white, indicating they were likely the original plasmid. The results suggest that the QuikChange^TM^ PCR is exponential and the product is linear with homologous ends.

### The QuikChange^TM^ PCR products are linear with homologous ends

**Figure 2.**
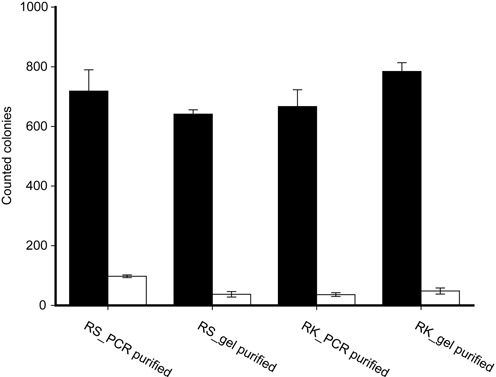
The products from the QuikChange^TM^ PCR and normal PCR generated similar numbers of mutant colonies. PCR reactions were done with the same pair of primers for site-directed mutagenesis with pBS-TAA as the template (RS) or for gene deletion with pBS-Kan as the template (RK). The PCR products were directly purified (PCR purified) or separated on agarose gel, extracted and purified (gel purified). Sixty nanograms of purified DNA was used for transformation. Black column, blue colonies; white column, white colonies. The data are averages of three experiments with standard deviations (error bar).

Several approaches were used to investigate whether the QuikChange^TM^ PCR products were linear with homologous ends. First, the PCR was done with the same primers but two different templates, pBS-TAA and pBS-Kan, to regenerate pBluescript SK-. The plasmids pBS-TAA and pBS-Kan had the insertion of a stop codon (TAA) and a Kan-resistant gene between the 18th and 19th codons of LacZα on pBluescript SK-, respectively (Supplementary Figure S3). When pBS-TAA was used as a template, the reaction was a typical QuikChange^TM^ PCR reaction, in which the primers annealed to the same region on the plasmid but in opposite directions. When pBS-Kan was used as the template, the reaction was a regular PCR reaction with the primers annealing to different locations separated by the Kan-resistant gene (Supplementary Figure S3). The two PCR yields were similar, according to gel electrophoresis analysis (Supplementary Figure S5). When the PCR products were directly purified or purified after gel electrophoresis and used to transform *E. coli* XL-1 Blue MRF’, they generated similar numbers of blue colonies, representing the conversion of pBS-TAA or pBS-Kan to pBluescript SK- (Figure 2). Five blue colonies from each sample were randomly chosen for sequencing, and they were all correctly mutated as expected. These results suggest that the PCR products originated from the two templates are essentially the same as linear DNA fragments with homologous ends.

Second, an end-filling test was done. The DpnI-treated PCR product was heated at 80°C to linearize any potential circular plasmids with staggered single strand DNA breaks, in which the 5′-overhangs had a calculated Tm of 72.4°C, and the ends were filled by the PCR DNA polymerase at 72°C. The sample with the end-filling step produced 1164 ± 101 blue colonies per 5 μl of the QuikChange^TM^ PCR product after transformation, and the percentage of blue colonies was 92.3% ± 0.8%. The control without the end-filling step produced 1025 ± 39 blue colonies, and the percentage of blue colonies was 93.4% ± 0.6%. The results were similar, suggesting that the QuikChange^TM^ PCR products are mainly linear DNA molecules with homologous ends.

Third, the QuikChange^TM^ PCR product was ligated to form the circular plasmid, and the ligation site was sequenced. The sequence revealed that the ligated region contained the inverted repeat of the two QuikChange^TM^ PCR primers (Supplementary Figure S6), demonstrating that the QuikChange^TM^ PCR product is linear with homologous ends.

Fourth, the *E. coli* RecET recombination system is known to enhance homologous recombination with short homologous ends (18). Thus, we tested whether an *E. coli* strain carrying the system could enhance recombination and forming more colonies when transformed with the QuikChange^TM^ PCR product. Strain GB05dir carries the *recET* genes under the control of an arabinose promoter on the genome, and strain GB05 is the parent strain (12). When 2 ng of intact pBluescript SK- was electroporated into the cells, the numbers of blue colonies for strains GB05 and GB05dir were 3.42 ± 0.57 × 10^5^ and 8.27 ± 2.32 × 10^5^, similar to each other. When 60 ng of the purified PCR product was electroporated into the cells, the numbers of blue colonies for strains GB05 and GB05dir were 3.92 ± 1.93 × 10^3^ and 1.26 ± 0.13 × 10^5^, respectively. The results suggest that GB05dir with the RecET system effectively recombine the linear PCR product with short homologous ends into the circular plasmid.

### Phusion uses partially overlapping primers to amplify the target plasmid

**Figure 3.**
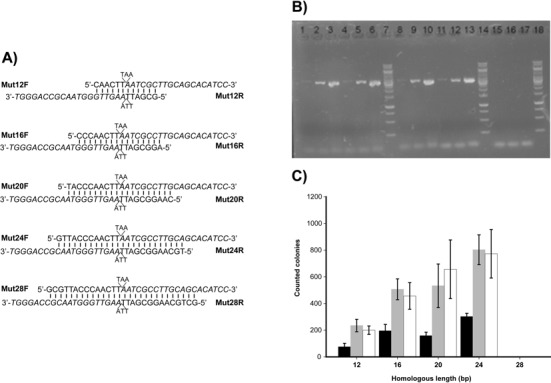
The Phusion-dependent site-directed mutagenesis with different lengths of the overlapping regions. (**A**) The primer pairs are shown with the reverse primers in reverse and overlapping regions aligned, and the mutation is for the deletion of TAA as indicated. (**B**) Gel electrophoresis of the PCR products produced by Phusion. The Phusion PCR was done in 20 μl with 2 ng of pBS-TAA as template and 0.5 μM partially overlapping primers in the Phusion HF buffer. The PCR was done with initial denaturation at 98°C for 3 min, followed by 16, 20 or 25 cycles of denaturizing at 98°C for 25 s, annealing at 69°C for 30 s and extension at 72°C for 90 s, and the final step was incubation at 72°C for 10 min. Three microliters of sample was analyzed. Lanes 1–3, PCR with primers containing 12 nucleotides in the overlapping region (Mut12F/R); 4–6, PCR with primers Mut16F/R; 8–10, PCR with primers Mut20F/R; 11–13, PCR with primers Mut24F/R; 15–17, PCR with primers Mut28F/R. For each primer pair: 1st lane, 16 cycles; 2nd lane, 20 cycles; 3rd lane, 25 cycles. Lanes 7, 14, 18, DNA markers. (**C**) The effects of the homologous lengths and PCR cycles on the mutation efficiency. The PCR was done as described in Figure 3B legend. After PCR and DpnI digestion, 5 μl of the PCR product was used for transformation. All blue colonies were counted. Black column, 16 PCR cycles; gray column, 20 PCR cycles; white column, 25 PCR cycles. The values were the averages of three results with standard deviations. The percentages of blue colonies ranged from 95 to 99%.

Phusion did not amplify pBS-TAA with the complementary primers mutF2 and mutR2 (Figure 1), but amplify pBS-TAA with partially overlapping primers (Figure 3B). The partially overlapping primers had a fixed length of 20 nucleotides for the forward and reverse primers after the mutation site at the 3′-ends and varied lengths before the mutation site at the 5′-ends to give an overlapping region of 12 to 24 nucleotides (Figure 3A). For example, in mut12F and mut12R, the overlapping region of 12 nucleotides was underlined, and the region after the mutation site was italicized, and the two regions shared six nucleotides that were both underlined and italicized (Figure 3A). The mutation was to delete the stop codon TAA in the overlapping region. The 20-μl PCR reaction contained 2 ng of pBS-TAA, instead of 20 ng, and performed as recommended for Phusion by the supplier. The products were digested with DpnI (Figure 3B), and colonies were formed after transformation (Figure 3C). Although the 12-basepair homologous ends were enough to obtain recombination and blue colonies, the 16-basepair homologous ends significantly increased the number of colonies. Further increase of the homologous length helped less significantly. The increase of PCR cycles was associated with the increase in amounts of the PCR products (Figure 3B) and the number of blue colonies (Figure 3C), but 20 cycles were sufficient. When the length of the overlapping regions increased to 28 nucleotides, no PCR products and colonies were detected (Figure 3). However, the formation of primer dimers was increased with the increased length of the overlapping region (Figure 3B, bottom), indicating that there is competition between formation of the perfectly matching primer dimer and the partially matching primer-template duplex, and the former becomes dominant when the overlapping region is significantly long.

Phusion and partially overlapping primers were used to mutate the *cas9* gene cloned into pTrc99a (pTrc99a_Cas9) containing 8289 base pairs. The Cas9 protein has two catalytic residues Asp10 and His480 (19,20), and they were mutated to Cas9 D10A and Cas9 H840A with the primer pair of Cas9-D10AF and Cas9-D10AR and the primer pair of Cas9-H840AF and Cas9-H840AR (Supplementary Table S2). About 120 colonies were obtained from each mutagenesis. Three colonies from each mutagenesis were selected for sequencing, and all had the correctly mutated sequences.

### The qPCR melting curve analysis of 5′-overhang filling

**Figure 4.**
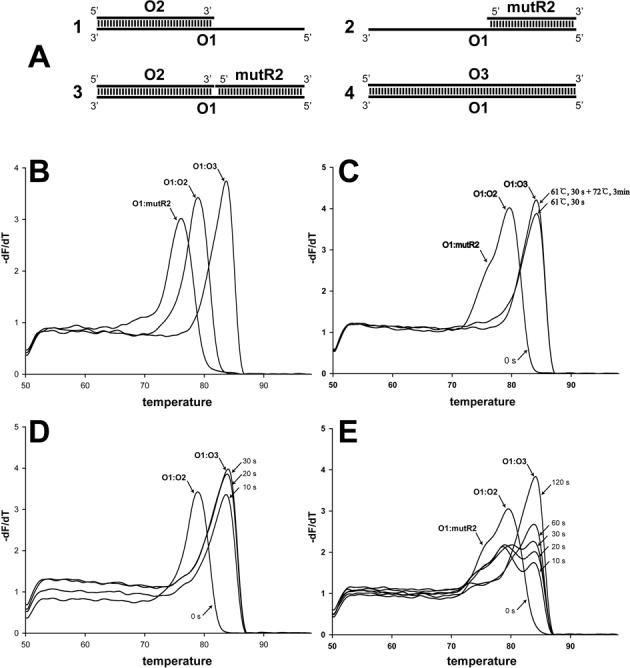
Testing 5′-overhang filling with the qPCR melting curve analysis. (**A**) The complementary relationships of oligonucleotides O1 (80 nucleotides), O2 (47 nucleotides), O3 (80 nucleotides) and mutR2 (33 nucleotides). (**B**) The oligonucleotide pairs of O1:mutR2, O1:O2 and O1:O3 at 0.4 μM were formed by heating and cooling in the PCR buffer, to which SYBR Green I was added, and analyzed by using the melting curve function of qPCR. (**C**) A typical PCR reaction was done with three oligonucleotides O1, O2 and mutR2 (Figure 4A) at 0.4 μM each. The reactions were stopped at 0 s, after annealing at 61°C, and after extension at 72°C by adding EDTA to 5 mM, and then SYBR Green I was added before melting curve analysis. (**D**) The (0.4 μM) O1:O2 complex (Figure 4A1) was formed by heating and cooling, and then PrimeStar was added to fill the 5′-overhang, producing O1:O3 at 61°C. (**E**) The (0.4 μM) O1:O2:MutR2 complex (Figure 4A3) was also formed and converted to O1:O3 (Figure 4A4) by PrimeStar at 61°C. Reactions were stopped by adding EDTA to 5 mM, and then SYBR Green I was added before melting curve analysis. The DNA duplexes were detected according to their Tm's.

The qPCR melting curve analysis was shown to be able to detect the Tm's of short DNA duplexes O1:mutR2, O1:O2 and O1:O3 under the testing conditions as 76.3, 78.9 and 83.4°C, respectively (Figure 4A and B). In a typical PrimeStar PCR reaction with three oligonucleotides O1, O2 and mutR2, O1 was the template and O2 was the primer. The product of primer extension was a short DNA duplex of 80 base pairs, the same as the duplex of O1 and O3 (80 nucleotide) (Figure 4A). After the denaturation and annealing at 61°C for 30 s, most of the O1:O2 complexes were converted to O1:O3 even in the presence of mutR2 (Figure 4C), which could anneal to the 5′-overhang of the O1:O2 complex (Figure 4A) and inhibit the primer extension. Further incubation at 72°C did not significantly increase the O1:O3 complex (Figure 4C) because the reaction was almost complete at 61°C. To test whether mutR2 could inhibit the primer extension, the O1:O2 and O1:O2:mutR2 complexes were formed by heating and cooling. Then, PrimeStar was added and the reaction was incubated at 61 or 72°C. In the absence of mutR2, the reaction was almost complete in 20 s at 61°C (Figure 4D) and in 10 s at 72°C (Supplemental Figure S8A). The presence of mutR2 inhibited the reaction, and the reaction was complete in 120 s at 61°C (Figure 4E) and in 30 s at 72°C (Supplemental Figure S8B).

## DISCUSSION

Some DNA polymerases, such as PfuTurbo, PrimeStar, KOD FX and Pyrobest, are able to use complementary primers to amplify the tested plasmid, but TransTaq, Phusion and Q5 cannot in our test. The failure is likely due to the high annealing temperatures for Phusion and Q5 that may promote the formation of the perfectly matching primer dimers over the formation of the primer-template duplex containing mismatches. It also could be due to the intrinsic nature of the enzyme, and the enzyme with a higher affinity to the substrate (primer-template duplex) has a better chance to stabilize the duplex before its dissociation. For example, the PrimeStar has high affinity to its substrate as described in its specification (Takara), and it can replace PfuTurbo in the QuikChange^TM^ PCR reaction with complementary primers. However, all of the enzymes can use partially overlapping primers to amplify the tested plasmid (Supplementary Figure S7). When compared side-by-side, the PCR with partially overlapping primers yielded more PCR products (Supplementary Figure S7). In general, the number of colonies was proportional to the amount of product produced.

**Figure 5.**
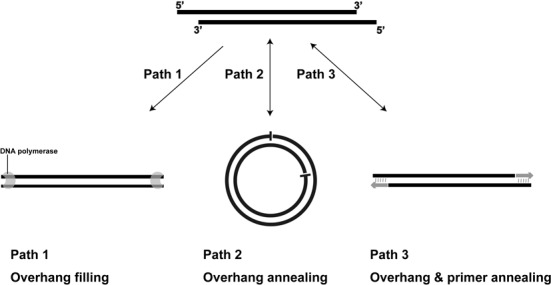
Three potential paths for the double-stranded DNA with 5′-overhangs during PCR. When complementary primers are used to amplify a circular plasmid, the initial products anneal, producing double-stranded DNA with 5′-overhangs. Three potential paths are presented. Path 1, the PCR enzyme fills the overhangs to generate linear DNA fragments with blunt ends, which can be used as the templates for subsequent PCR amplification. Path 2, the overhangs self-anneal to form circular DNA with single-stranded breaks. Path 3, the overhangs anneal to primers to produce linear DNA with single-stranded breaks. Path 1 is irreversible, and Paths 2 and 3 are reversible.

Our experimental data suggested that the QuikChange^TM^ PCR products are linear, double-stranded DNA molecules with short homologous ends. It is likely that the linear DNA molecules from the first round of PCR anneal to form linear double-stranded DNA with 5′-overhangs (Figure 5), and the PCR enzyme fills in the overhangs to generate linear DNA molecules containing blunt ends (Figure 5, Path 1), which are the templates for subsequent PCR amplifications. Theoretically, the linear double-stranded DNA with 5′-overhangs could anneal to each other, forming circular plasmid DNA with single-stranded breaks (Figure 5, Path 2), or could anneal to the primers (Figure 5, Path 3). In our experiments with the primers mutF2 and mutR2 for PCR, the annealing temperature was 61°C and extension temperature was 72°C. The calculated Tm of the primers is 72.4°C, at which the annealing and dissociation rates of the primer-template complex are equal. At temperatures close to the Tm, some of the products of Paths 2 and 3 (Figure 5) dissociate, and the released linear DNA molecules with 5′-overhangs are filled by the PCR enzyme (Figure 5, Path 1). Since Path 1 is irreversible, it removes the linear DNA with 5′-overhangs, driving further dissociation of the products of Paths 2 and 3 (Figure 5). The process should lead all linear DNA with 5′-overhangs been filled during PCR (Figure 5, Path 1).

We tested the model (Figure 5) with a three-oligonucleotide system, in which O1 and O2 anneal to form a short DNA duplex with a 5′-overhang (Figure 4A). The primer mutR2 was chosen to bind to the 5′-overhang of the O1:O2 duplex (Figure 4A) because the primer pair mutF2 and mutR2 was used for the conversion of pBS-TAA to pBluescript SK-. Even at the annealing temperature (61°C), Path 1 (end-filling) and the reverse reactions of Paths 2 and 3 occurred (Figure 4). Our data showed that within a single PCR cycle, the 5′-end was filled, and most filling was done at 61°C (Figure 4C). At both 61 and 72°C, the reverse reactions of (Figure 5) Paths 2 and 3 occurred and the released DNA duplex with 5′-overhang was filled by the enzyme (Figure 4D and E and Supplementary Figure S8A and B). The tested concentration of O1:O2 at 0.4 μM is much higher than the double-stranded DNA with 5′-overhangs after the first cycle of the QuikChange^TM^ PCR. Under ideal conditions, the double-stranded DNA concentration after the first cycle equals to the template DNA concentration, which is calculated to be 0.5 nM for 20 ng of pBluescript SK- in 20 μl. Thus, the double-stranded DNA with 5′-overhangs should be completely filled in the next cycle during annealing at 61°C or during extension at 72°C.

**Figure 6.**
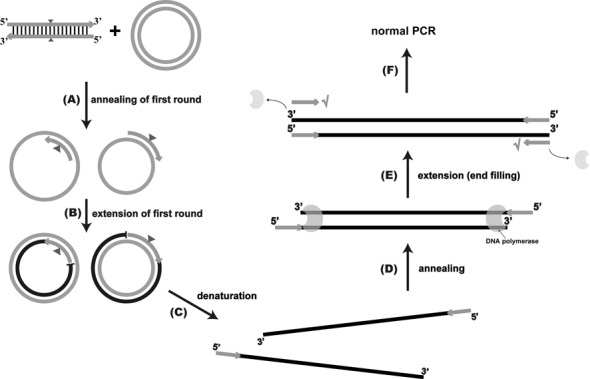
Schematic presentations of the revised PCR amplification of the QuikChange^TM^ protocol. (**A**) The primers are annealed to the template DNA. (**B**) The products of the first round of PCR are the same length as the plasmid. (**C**) In the following rounds of PCR, the newly synthesized strands are denatured and separated from the plasmid template. (**D**) Then, the new strands are annealed to form double-stranded DNA molecules with 5′-overhangs. (**E**) The overhangs were filled by the PCR enzyme. (**F**) The double-stranded linear DNA with homologous ends is then used as the template for subsequent PCR, and ‘√’ indicates that the QuikChange^TM^ PCR primers can be used to amplify the fragment. The gray cycles represent the parental plasmid DNA. The black lines or circles represent the DNA amplified using the parental DNA as the template. Gray arrows indicate the QuikChange^TM^ PCR primers. Gray triangles indicate the location of the mutation.

Figure 6 summarizes the QuikChange^TM^ PCR process to generate linear DNA molecules with homologous ends. After the end filling (Figure 5, Path 1), the DNA fragment with blunt ends is used as the template for exponential PCR amplification, producing more copies of the DNA fragment with homologous ends. When the PCR product is transformed into *E. coli*, the PCR product is joined through homologous recombination to form the plasmid with the desired mutation. Our findings are consistent with the *E. coli*'s ability to combine short homologous ends via recombination, and the ability has been used for cloning (11,21). Since *E. coli* XL-1 Blue MRF’ is a *recA* mutant, the recombination for the QuikChange^TM^ products is through the uncharacterized RecA-independent system. The *E. coli*'s ability can be enhanced when it expresses the RecET system (18). This was confirmed with the *E. coli* strain GB05dir expressing the *recET* genes (12) that produced 32 times more colonies than its parent strain GB05 after electroporation.

The new QuikChange^TM^ mechanism has several implications. First, partially overlapping primers can be used to obtain the PCR product with homologous ends. The strategy has been successfully used to modify the QuikChange^TM^ method by several groups (4,6,8–10). However, the use of partially overlapping primers is incompatible with the proposed mechanism. Our findings support the use of partially overlapping primers because the products are the same whether completely or partially overlapping primers are used. The partially overlapping primers may be advantageous to minimize the formation of primer dimers. Second, the partially overlapping primers allow the use of all tested enzymes (Supplemental Figure S7), including Phusion, for PCR. Third, a small amount of template DNA is required for exponential amplification with partially overlapping primers, minimizing the template DNA surviving the DpnI digestion and yielding colonies containing the template plasmid. Fourth, a successful PCR is the foundation for the mutagenesis and the PCR product should be detectable. Fifth, *E. coli* has the intrinsic capability to recombine short homologous ends via a RecA-independent recombination system, and the efficiency can be significantly enhanced by using *E. coli* GB05dir expressing the *recET* genes (12).

With the new understanding, we tested Phusion with partially overlapping primers and a reduced amount of the template DNA. The results were as expected (Figure 3), but the primer pair of Mut28F and Mut28R did not work for both PCR and mutagenesis. The failure of Phusion to use complementary primers and primers with a long overlapping region in our experiments is likely due to the higher annealing temperature for Phusion PCR, which may favor the primer dimer formation (Figure 3B, bottom) instead of primer annealing to the template with mismatches.

A simple site-directed mutagenesis method using Phusion is developed. The method uses partially overlapping primers. The partially overlapping primers are about 30 nucleotides in length with approximately 20 nucleotides in the overlapping region and about 10 nucleotides in the non-overlapping region at the 3′-ends. The mutation site is preferentially at the middle of the overlapping region. The PCR is done in 20 μl volume with 2 ng of template plasmid DNA for 20 cycles with conditions as recommended for Phusion. We obtained PCR products with annealing temperatures from 67 to 72°C, suggesting the PCR conditions can tolerate some variations from the recommended annealing temperature. After DpnI digestion, 5 μl of the PCR product is transformed into *E. coli* XL-1 Blue MRF’ to obtain the mutant plasmid. Alternatively, electroporation can be used. *E. coli* GB05dir can be used when more recombinant colonies are desirable.

The new mechanism and method allow routine site-directed mutagenesis with commercially available reagents, and the method largely becomes a PCR issue. When PCR products are detected, both transformation and electroporation can be used to deliver the PCR products into *E. coli* cells for recombination. The advantages of using partially overlapping primers for the QuikChange^TM^ method have previously been recognized (4,6,8–10); however, their use is incompatible with the proposed mechanism of the QuikChange^TM^ method. Here we provide evidence that the QuikChange^TM^ mutagenesis proceeds through exponential amplification *in vitro* and homologous recombination *in vivo* with either completely or partially overlapping primers. Our findings provide the theoretical validation for using partially overlapping primers. When using Phusion for the PCR, we recommend using partially overlapping primers. Phusion offers several advantages over PfuTurbo, being faster, more robust and with higher fidelity. Both New England BioLabs and Thermo Fisher Scientific offer Phusion-based site-directed mutagenesis kits, which use non-overlapping primers for PCR, and the PCR products are ligated by DNA ligase before transformation. Our protocol does not need a ligation step.

## SUPPLEMENTARY DATA

Supplementary Data are available at NAR Online.

SUPPLEMENTARY DATA
