# Supplementary material for: New insights into the QuikChangeTM process guide the use of Phusion DNA polymerase for site-directed mutagenesis
Source: Nucleic Acids Res. 2014 Nov 15;43(2):e12. doi: 10.1093/nar/gku1189 (PMC4333370; doi:10.1093/nar/gku1189)
Supplement: SUPPLEMENTARY DATA [file supp_43_2_e12__index.html]

New insights into the QuikChangeTM process guide the use of Phusion DNA polymerase for site-directed mutagenesis — New insights into the QuikChangeTM process guide the use of Phusion DNA polymerase for site-directed mutagenesis — SUPPLEMENTARY DATA 

# New insights into the QuikChangeTM process guide the use of Phusion DNA polymerase for site-directed mutagenesis

## SUPPLEMENTARY DATA

**Files in this Data Supplement:**

- SUPPLEMENTARY DATA
