# Supplementary material for: New insights into the QuikChangeTM process guide the use of Phusion DNA polymerase for site-directed mutagenesis
Source: Nucleic Acids Res. 2014 Nov 15;43(2):e12. doi: 10.1093/nar/gku1189 (PMC4333370; doi:10.1093/nar/gku1189)
Supplement: SUPPLEMENTARY DATA [file supp_gku1189_nar-01758-met-g-2014-File008.docx]

**New insights into the QuikChange^TM^ process guide the use of Phusion DNA polymerase for site-directed mutagenesis**

Yongzhen Xia^1^, Wenqiao Chu^1^, Qingsheng Qi^1^, Luying Xun^1,2^*

^1^State Key Laboratory of Microbial Technology, Shandong University, Jinan, 250100, P. R. China

^2^School of Molecular Biosciences, Washington State University, Pullman, WA 99164-7520, USA

Contents

1. Supplementary Tables

Supplementary Table S1 - The bacterial strains and plasmids

Supplementary Table S2 - The primers used in this study

1. Supplementary Figures

Supplementary Figure S1 - Schematic presentation of the PCR amplification and mutagenesis shown in the QuikChange^TM^ manual

Supplementary Figure S2 - The PCR programs utilized in the experiment for the test of different DNA polymerases

Supplementary Figure S3 - The scheme of mutating pBS-TAA and pBS-Kan back to pBluescript SK-

Supplementary Figure S4 - Gel electrophoresis of the PCR products produced by using different samples from a modified single primer method

Supplementary Figure S5 - Gel electrophoresis of the PCR products produced from different templates

Supplementary Figure S6 - The sequencing chromatogram covering the ligated region of the QuikChange^TM^ PCR product with pBS-TAA as the template

Supplementary Figure S7 - Gel electrophoresis of the PCR products produced by different DNA polymerases

Supplementary Figure S8 - The melting curve analysis of the O1:O2 production at 72°C

3- Supplementary References

**Supplementary Table S1. The bacterial strains and plasmids**

| Strains and plasmids | Functions and features | Reference |
| --- | --- | --- |
| **Strains** |  |  |
| *E. coli* XL1 Blue MRF’ | Δ(*mcrA*)*183* Δ(*mcrCB-hsdSMR-mrr*)*173 endA1 supE44 thi-1 recA1* gyrA96 relA1 lac*[F′*proAB lacI^q^*ZΔ*M15*Tn*10*(Tet^r^)]* | Agilent |
| *E. coli* GB05 | Derived from DH10B, *fhuAIS2*; Δ*recET*, Δ*ybcC* | (12) |
| *E. coli* GB05dir | Derived from DH10B, *fhuAIS2*; Δ*recET*, *ybcC*:: pBAD *abgA* | (12) |
| **Plasmids** |  |  |
| pBluescript SK^-^ | A normal cloning vector, ampicillin resistance, Amp^+^ | Stratagene |
| pBS-Kan | A kanamycin resistance gene from pBBR1MCS2 was inserted into the same region of pBS-TAA to stop the expression of LacZα. | This study |
| pBS-TAA | A stop codon was inserted into the *lacZα* gene of pBluescript SK- to stop the expression of LacZα. | This study |
| pTrc99a-Cas9 | The *cas9* gene was cloned in the expression vector pTrc99a. | This study |

**Supplementary Table S2. The primers used in this study**

| NO. | Primers | Sequences | Purposes and characteristics |
| --- | --- | --- | --- |
| **Used for Plasmids construction and Quikchange^TM^ SDM reactions** | | | |
| 1 | mutF1* | CTGGCGTTACCCAACTTtaa*AATCGCCTTGCAGCAC* | Front primer of QuikChange^TM^ SDM reaction to get pBS-TAA |
| 2 | mutR1* | GTGCTGCAAGGCGATTtta*AAGTTGGGTAACGCCAG* | Reverse primer of QuikChange^TM^ SDM reaction to get pBS-TAA |
| 3 | pBSF | AAGTTGGGTAACGCCAGGGT | Used for pBS-Kan plasmid construction |
| 4 | pBSR | AATCGCCTTGCAGCACATCC | Used for pBS-Kan plasmid construction |
| 5 | KanF | ACCCTGGCGTTACCCAACTTATCGGGATATGCAGGCCAAGG | Used for pBS-Kan plasmid construction |
| 6 | KanR | GGATGTGCTGCAAGGCGATTCATCAGCACCTTGTCGCCTTG | Used for pBS-Kan plasmid construction |
| 7 | mutF2* | CTGGCGTTACCCAACTT*AATCGCCTTGCAGCAC* | Used to mutate pBS-Kan and pBS-TAA back to pBluescript SK- |
| 8 | mutR2* | GTGCTGCAAGGCGATT*AAGTTGGGTAACGCCAG* | Used to mutate pBS-Kan and pBS-TAA back to pBluescript SK- |
| 9 | mutF3* | CTGGCGTTACCCAACTT*AATCGCCTTGCAGCAC* | Same primer to #7, but its 5' end is phosphorylated |
| 10 | mutR3* | GTGCTGCAAGGCGATT*AAGTTGGGTAACGCCAG* | Same primer to #8, but its 5' end is phosphorylated |
| 11 | mut12F | CAACTT*AATCGCCTTGCAGCACATCC* | Front primer used in the modified QuikChange^TM^ site-directed mutagenesis 12 bp homologous ends |
| 12 | mut12R | GCGATT*AAGTTGGGTAACGCCAGGGTTTTC* | Reverse primer used in the modified QuikChange^TM^ site-directed mutagenesis with 12 bp homologous ends |
| 13 | mut16F | CCCAACTT*AATCGCCTTGCAGCACATCC* | Front primer used in the modified QuikChange^TM^ site-directed mutagenesis 16 bp homologous ends |
| 14 | mut16R | AGGCGATT*AAGTTGGGTAACGCCAGGGTTTTC* | Reverse primer used in the modified QuikChange^TM^ site-directed mutagenesis with 16 bp homologous ends |
| 15 | mut20F | TACCCAACTT*AATCGCCTTGCAGCACATCC* | Front primer used in the modified QuikChange^TM^ site-directed mutagenesis 20 bp homologous ends |
| 16 | mut20R | CAAGGCGATT*AAGTTGGGTAACGCCAGGGTTTTC* | Reverse primer used in the modified QuikChange^TM^ site-directed mutagenesis with 20 bp homologous ends |
| 17 | mut24F | GTTACCCAACTT*AATCGCCTTGCAGCACATCCC* | Front primer used in the modified QuikChange^TM^ site-directed mutagenesis 24 bp homologous ends |
| 18 | mut24R | TGCAAGGCGATT*AAGTTGGGTAACGCCAGGGTTTTC* | Reverse primer used in the modified QuikChange^TM^ site-directed mutagenesis with 24 bp homologous ends |
| 19 | mut28F | GCGTTACCCAACTT*AATCGCCTTGCAGCACATCCC* | Front primer used in the modified QuikChange^TM^ site-directed mutagenesis with 28 bp homologous ends |
| 20 | mut28R | GCTGCAAGGCGATT*AAGTTGGGTAACGCCAGGGTTTTC* | Reverse primer used in the modified QuikChange^TM^ site-directed mutagenesis with 28 bp homologous ends |
| 21 | Cas9-D10AF | ATAGGCTTAGc*TATCGGCACAAATAGCGTCGG* | Front primer used to mutate pCas9 plasmid, according to the modified QuikChange^TM^ site-directed mutagenesis method |
| 22 | Cas9-D10AR | TGTGCCGATAg*CTAAGCCTATTGAGTATTTC* | Reverse primer used to mutate pCas9 plasmid, according to the modified QuikChange^TM^ site-directed mutagenesis method |
| 23 | Cas9-H840AF | GATGTCGATgc*CATTGTTCCACAAAGTTTCC* | Front primer used to mutate pCas9 plasmid, according to the modified QuikChange^TM^ site-directed mutagenesis method |
| 24 | Cas-H840AR | GGAACAATGgc*ATCGACATCATAATCACTT* | Reverse primer used to mutate pCas9 plasmid, according to the modified QuikChange^TM^ site-directed mutagenesis method |
| 25 | O1 | CTGGCGTTACCCAACTTAATCGCCTTGCAGCACCCCTCAATATGGCAAATGCACGTTTAATAGGCGCCCGTCTACACCGG | Oligo designed for 5’-overhang filling test |
| 26 | O2 | CCGGTGTAGACGGGCGCCTATTAAACGTGCATTTGCCATATTGAGGG | Oligo designed for 5’-overhang filling test |
| 27 | O3 | CCGGTGTAGACGGGCGCCTATTAAACGTGCATTTGCCATATTGAGGGGTGCTGCAAGGCGATTAAGTTGGGTAACGCCAG | Complementary to O1 |

**Note**: Mutation positions were written in lower case. The reverse primers with the completely complementary sequences to the corresponding forward primers were marked with asterisk. Letters with underline represent homologous sequences for recombination. The pairing region with template was italicized.


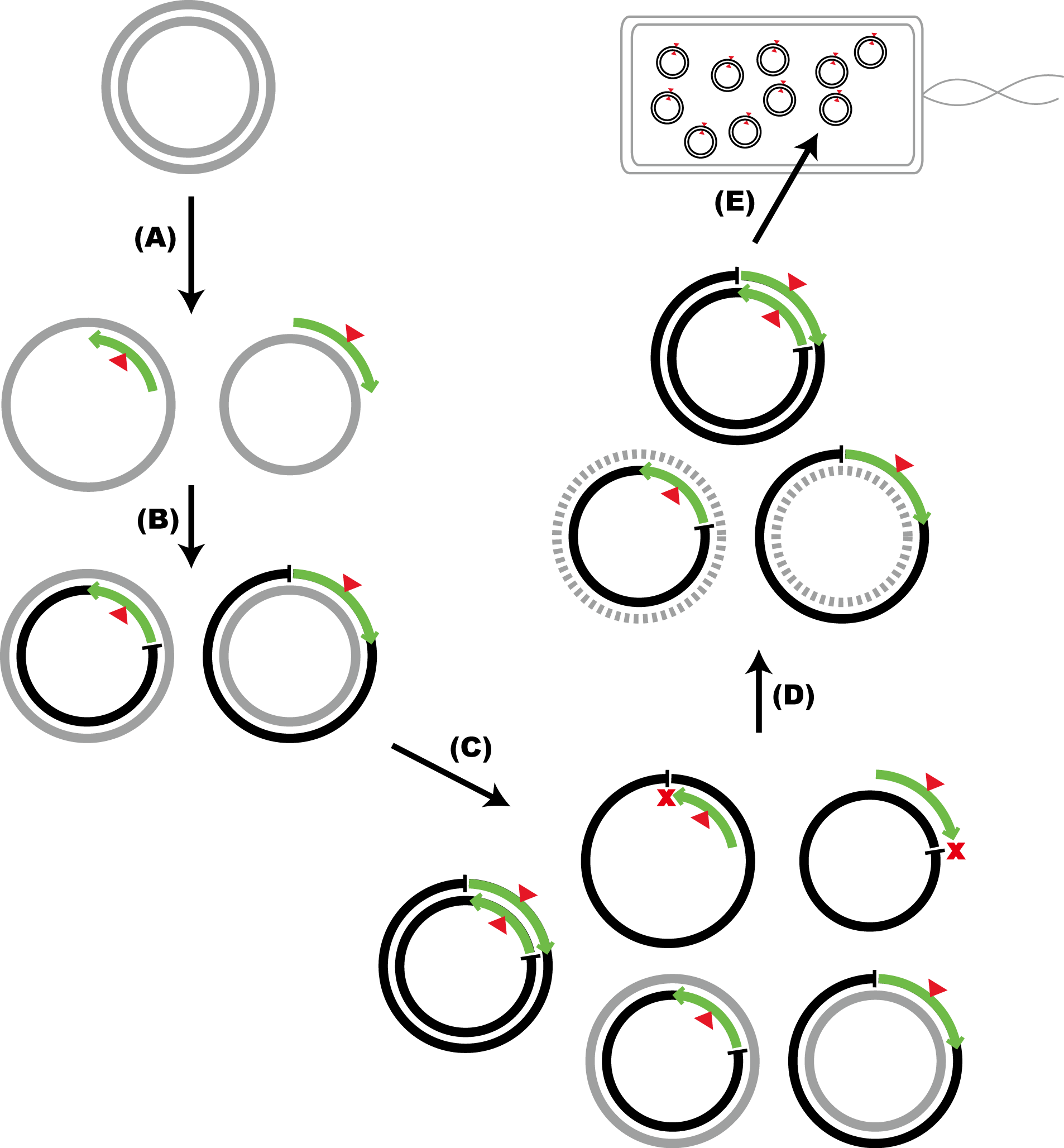


**Supplementary Figure S1. Schematic presentation of the PCR amplification and mutagenesis shown in the** **QuikChange^TM^ manual.** A) The primers are annealed to circular methylated DNA; B) In the first round of PCR, the primers extend by using parental plasmid as template; C) PCR extension fails when primers annealed to newly synthesized “nicked” DNA noted in the QuikChange^TM^ protocol where is marked with red ‘×’; D) The methylated template DNA is digested by DpnI; E) The SDM PCR products are transformed into *E. coli* competence cells, and cycle DNA products with two staggered nicks are repaired. The grey cycles represent the parental plasmid DNA, the cycles of black lines represent the synthesized DNA, and the cycles of grey dash line are the methylated parental DNA degraded by DpnI. Green arrows indicate the primers; Red Triangles indicate the location of the mutations/deletions/insertions; Short bars indicate the “nicks” in the newly-synthesized plasmid.


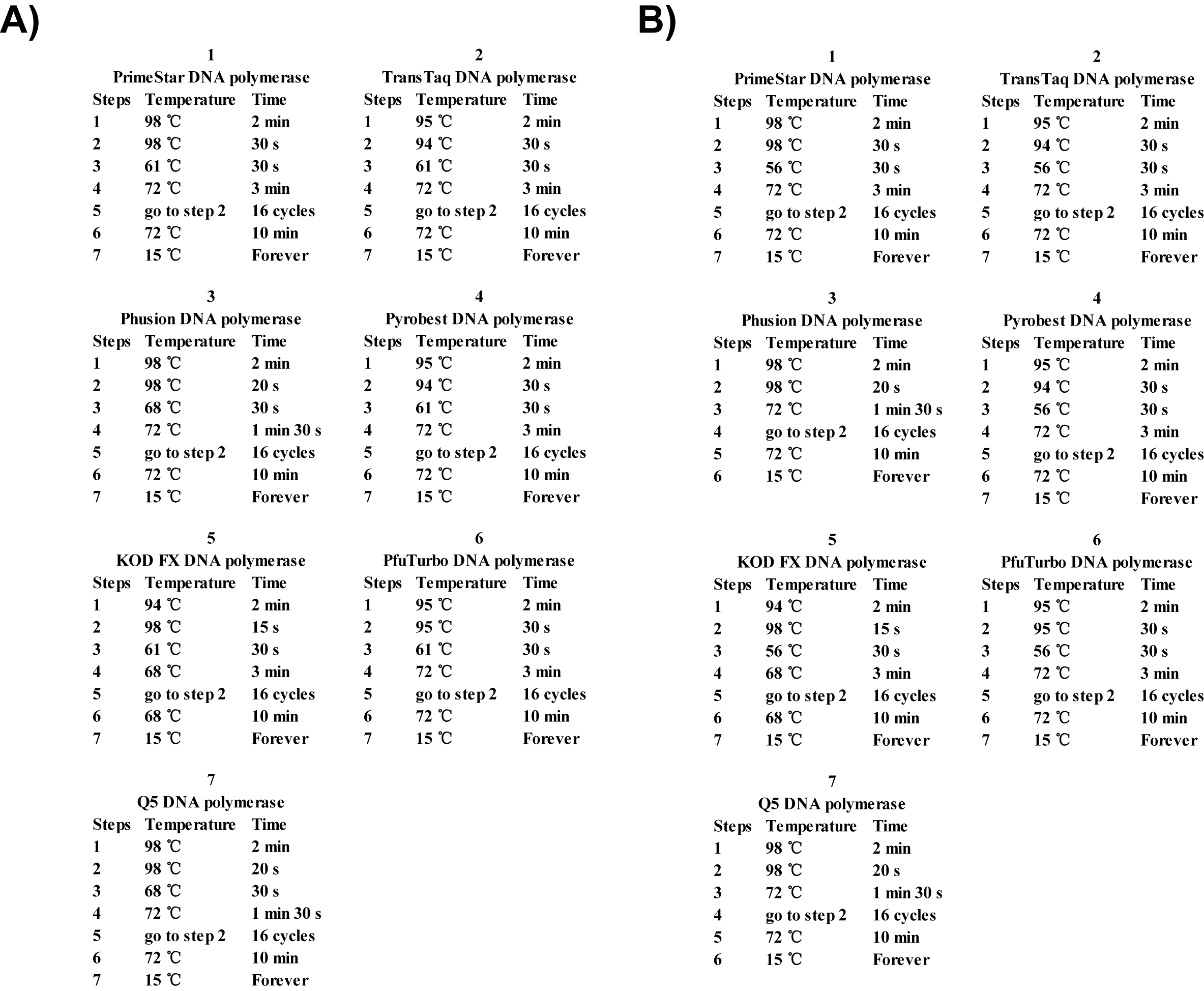


**Supplementary Figure S2. The PCR programs utilized in the experiment for the test of different DNA polymerases.** A) The PCR programs were utilized for pair of mutF2 and mutR2 primers (completely overlap primers); B) The PCR programs were utilized for pair of mut20F and mut20R primers (partly overlap primers).

**
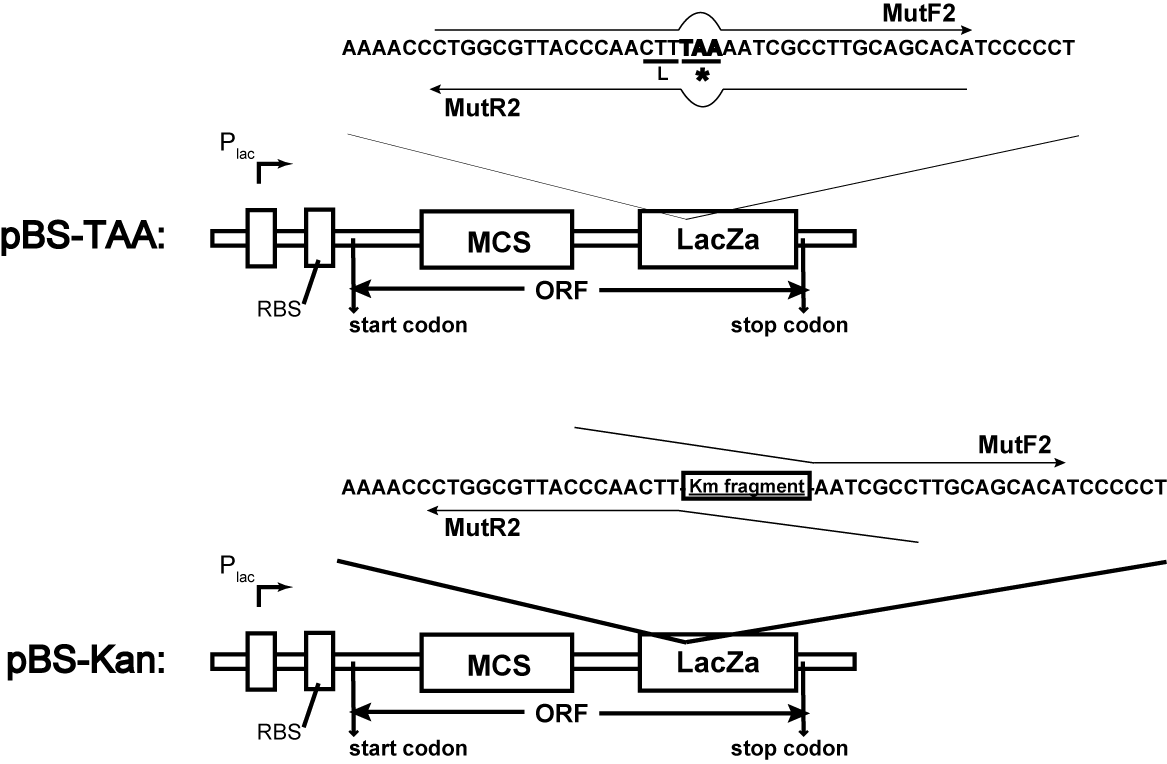
**

**Supplementary Figure S3. The scheme of mutating pBS-TAA plasmid and pBS-Kan back to pBluescript SK-**

**
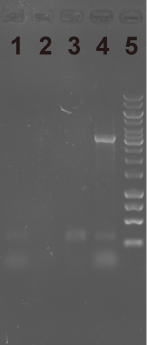
**

**Supplementary Figure S4. Gel electrophoresis of the PCR products produced by using different samples from a modified single primer method.** Five μl of sample was analyzed. Samples in lanes 1, 2, and 3, PCR was done with either Mut20F or Mut20R the single primer. Lane 1, the 1:1 (volume) mixed product after annealing; Lane 2, the PCR product with Mut20R as the primer; Lane 3, the PCR product was produced with Mut20F as the primer; Lane 4, the PCR product was obtained by using the primer pair, which was a control. All PCR reaction used 20 ng/20 μl pBS-TAA as template and the same PCR procedure. The single primer method worked when the template plasmid DNA was increased from 20 ng to 400 ng and PCR cycles from 16 to 30 as suggested by a single-primer site-directed mutagenesis protocol (20). The number of blue colonies from the single primer PCR group was 139±34 (average of three experiments with standard deviation), and the percentage of blue colonies from the single primer PCR group was 80.3%±1.4%, possibly due to inefficient digestion by DpnI with the high concentration of template plasmid DNA.

**
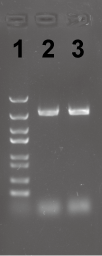
**

**Supplementary Figure S5. Gel electrophoresis of the PCR products produced from different templates.** Three µl of sample was analyzed. Lane 1, Trans5K DNA marker (TransGen Biotech, Beijing); Lane 2, PCR product with pBS-TAA as the template; Lane 3, PCR product with pBS-Kan as the template. The same molar concentration of templates was used for PCR.


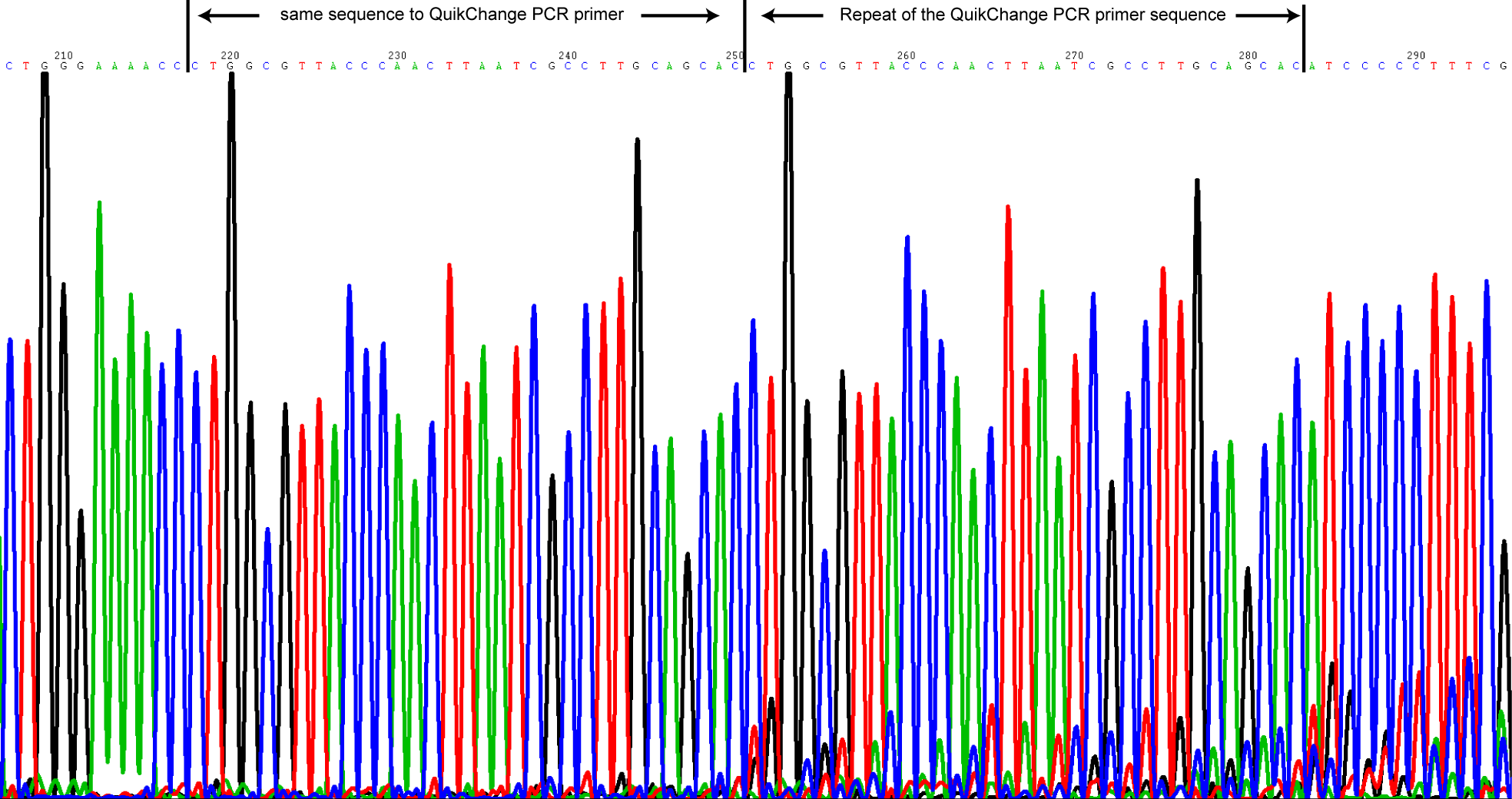


**Supplementary Figure S6. The sequencing chromatogram covering the ligated region of the QuikChange^TM^ PCR product with pBS-TAA as the template. The** QuikChange^TM^ PCR product was purified, ligated and then treated by DpnI. The ligation region was amplified and sequenced. The dominant signal peaks are the two primers joined at the 5’-ends, forming a short inverted repeat. The weak signal peaks that start at the middle of the inverted repeat reveal a sequence with the deletion of a single base, possibly due to some primers with a base missing at the 5’-ends. Careful examination did not show any possible sequence from the ligation of the overhang annealing (Fig. 4, Path 1).


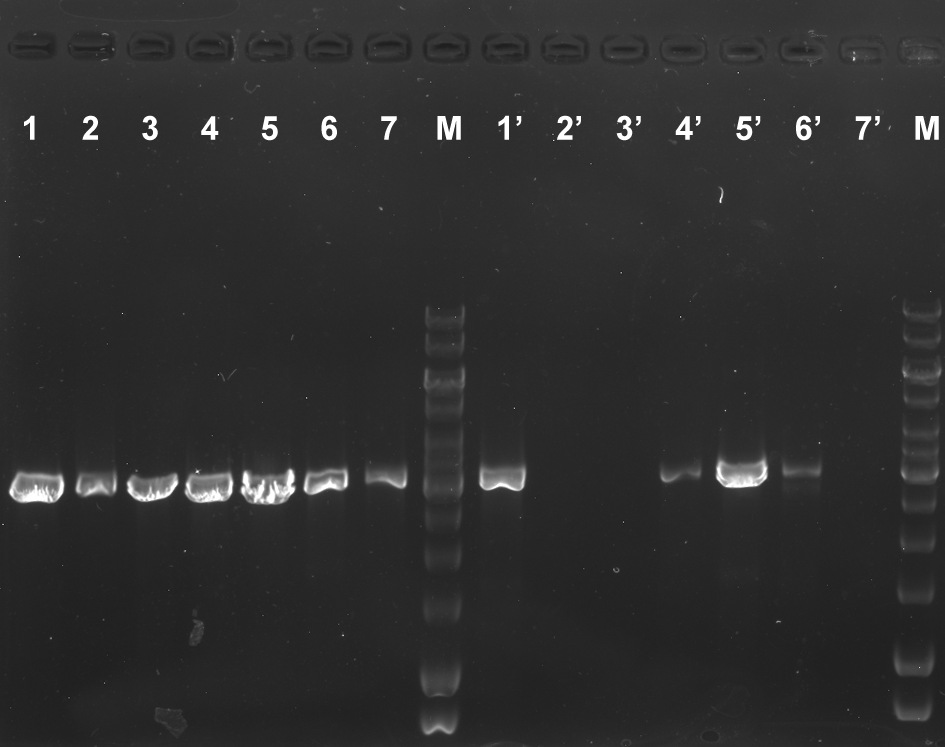


**Supplementary Figure S7 Gel electrophoresis of the PCR products produced by different DNA polymerases.** Three µl of each product was analyzed. The PCR products of lane 1-7 were obtained with mut20F and mut20R primers (partly overlap primers). The PCR products of lane 1’-7’ were obtained with mutF2 and mutR2 primers (completely overlap primers). Lane 1 and 1’, PCR products of PrimeStar; Lane 2 and 2’, PCR products of TransTaq; Lane 3 and 3’, PCR products of Phusion, Lane 4 and 4’, PCR products of Pyrobest; Lane 5 and 5’, PCR products of KOD FX polymerase, Lane 6 and 6’, PCR products of PfuTurbo; Lane 7 and 7’, PCR products of Q5 DNA polymerase.

**
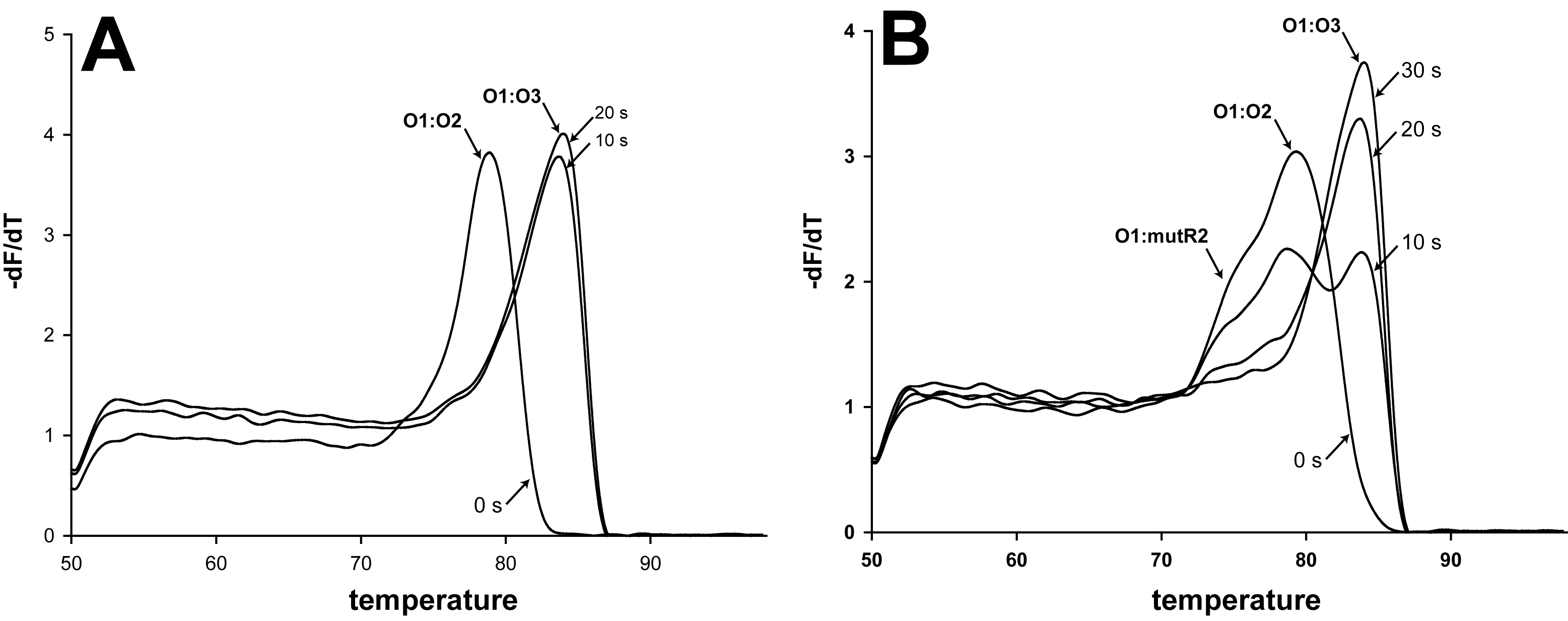
**

**Supplementary Fig. S8. The melting curve analysis of the O1:O2 production at 72**°C**.** The reactions were done essentially the same as described in Fig. 4D &E legend except at 72°C instead of 61°C. A) The conversion of 0.4 μM O1:O2 complex by PrimeStar to O1:O3 at 72°C. B) The conversion of 0.4 μM O1:O2:MutR2 complex by PrimeStar to O1:O3 at 72°C. Reactions were stopped at 0, 10, 20, 30 s by adding EDTA to 5 mM, and then SYBR Green I was added before melting curve analysis.

**Supplementary References**

**12. Fu, J., Bian, X., Hu, S., Wang, H., Huang, F., Seibert, P.M., Plaza, A., Xia, L., Muller, R., Stewart, A.F. *et al.* (2012) Full-length RecE enhances linear-linear homologous recombination and facilitates direct cloning for bioprospecting. *Nat Biotechnol*, 30, 440-446.**

**20. Edelheit, O., Hanukoglu, A. and Hanukoglu, I. (2009) Simple and efficient site-directed mutagenesis using two single-primer reactions in parallel to generate mutants for protein structure-function studies. *BMC Biotechnol*, 9, 61.**
